# Equine Stomach Development in the Foetal Period of Prenatal Life—A Histological and Histometric Study

**DOI:** 10.3390/ani12213047

**Published:** 2022-11-06

**Authors:** Dominik Poradowski, Aleksander Chrószcz

**Affiliations:** Department of Biostructure and Animal Physiology, Division of Animal Anatomy, Faculty of Veterinary Medicine, Wroclaw University of Environmental and Life Sciences, Kożuchowska 1, 51−631 Wrocław, Poland

**Keywords:** horse, stomach, development, foetal period, histometric

## Abstract

**Simple Summary:**

The prenatal development of equine stomach has been rarely elaborated. The majority of accessible literature focused on the embryonal period (ca. to the 45–50th day of gestation). The histological study of the stomach wall, including the metric measurements and the gastric gland development, filled the lack of detailed information about the processes taking place in more advanced periods of pregnancy (the foetal period). The achieved results showed that the growth rate of subsequent layers of the stomach wall provided differences comparing with the isometric growth rate of whole foetus length (CRL). The blind ventricular sac, the plicated edge margin, and the pyloric part growth rates were lower than CRL increase. The body of stomach showed a higher growth rate than the whole foetus length. The non-glandular and glandular part of gastric mucosa was distinguishable from the beginning of foetal period. The gastric glands developed the most rapidly in the body of stomach, especially in the late pregnancy. The parietal cells were visible in the gastric glands in the middle of foetal period and the chief cells could be identified in the late pregnancy. The dynamic processes occurring in the prenatal life did not finish in the moment of birth, but postnatally.

**Abstract:**

Histological and morphometrical analysis of the stomach wall was performed during the foetal period divided into three age groups (4th–11th month of gestation). The material was taken from non-glandular (the blind ventricular sac) and glandular parts (the plicated edge margin/cardiac part, the body of stomach and the pyloric part) of the stomach. It was preserved and prepared according to the standard protocol. The histological slides were stained (H-E, Masson-Goldner and PAS). The analyses were performed using the light microscope. All measurements were statistically elaborated. The crown-rump length growth rate was estimated as isometric. The blind ventricular sac growth rate was lower than CRL (negative allometric) and the partition of stomach mucosa into non-glandular and glandular part occurred in the 1st age group. The plicated edge margin/cardiac part and the pyloric part shoved similar tendencies. Only the body of stomach demonstrated a higher growth rate than CRL (positive allometric), which can be explained due to the strongest development of fundic glands. Moreover, comparing the adult reference group to the three parts of the foetal period, all metric values were lower than those achieved prenatally. The blind ventricular sac was covered with the multiple plane epithelium. The glandular parts of stomach that formed the superficial concave areas were covered with the simple columnar epithelium in the 1st age group, which developed to the cardiac, fundic, and pyloric glands in the 2rd and 3rd age groups. The *propria mucosae* was built with the mesenchyme, which differentiated later to the loose connective tissue. The muscular layer of mucosa was not clearly distinguishable in the 1st age group. The muscular layer of the stomach wall was formed with myoblasts in the 1st age group and later in the 2nd and the 3rd age groups built with fusiform myocytes divided into internal and external layers. The non-differentiated cells of glandular epithelium transformed into the parietal and chief cells. The first were visible in the gastric glands of the 2nd age group. Both of them were present in the 3rd age group gastric mucosa. The PAS staining proved a moderate PAS-positive reaction in the 2rd age group, while it was estimated as intense Pas-positive in the gastric glands in the 3rd age group and was comparable to postnatal observation (the adult reference group).

## 1. Introduction

Studies on domestic animal embryology have been frequently undertaken [1,2,3,4,5,6,7,8,9,10,11,12,13,14], but usually the scientific interest has focused on domesticated pig and ruminants, and papers devoted to horse embryology are rare [15]. Moreover, prenatal development of the alimentary tract of laboratory animals and its postnatal morphology have been studied for years [16,17,18,19,20,21,22,23,24,25,26].

The prenatal life can be divided into an embryonic period and a foetal period. The latter starts with sexual differentiation and gonad development [27]. Franciolli et al. [28] stated that equine foetal period starts on ca. 40th day of gestation even though the foetus sex remains undistinguishable until 47th day of gestation. The terminal part of the foregut initially forms a simple fusiform and tubular enlargement on 25th day of gestation, which is in fact the stomach primordium [28,29]. Next, rotation of the stomach brings the organ into its final position transverse to the long body axis. The stomach wall is differentiated into mucosa, submucosa, muscular layer, and serosa on 30th day of gestation [29].

The adult equine stomach includes a nonglandular, proventricular part (ventricular blind sac, *saccus caecus*) covered with multistratified plane epithelium and separated from a glandular part of the stomach mucosa with plicated edge margin (*margo plicatus*) [30]. Equine stomach is classified as a complex stomach, intermediate between a monogastric and a polygastric one. The gastric mucosa lacking any glands is detectable on 35th day of gestation, and the primordial organ is suspended between both gastric mesenteries on day 50 of gestation [29]. Until now, the exact time of the gastric glands development and specialized glandular cells presence in the horse foetal stomach remains unknown. Similar studies carried out for swine stomach during the foetal period brought about a detailed description of gastric gland prenatal development in swine foetuses [12]. Therefore, studies on stomach development in the foetal period are not only needed, but they can also enrich our knowledge on the embryology and developmental anatomy of horses, and serve as starting points for further interspecies comparisons.

Previous papers on horse embryology were general studies on foetus age estimation [31] or presented an early phase of prenatal life (15th to 107th day of gestation) [28,29]. The aim of this study was to describe the prenatal development of the stomach wall in foetal period (from the 4th to 11th month of gestation). Chrószcz [13] studied the morphometry and topography of a swine stomach in the foetal period between 35th and 114th day of gestation. An earlier study by Poradowski and Chrószcz [15] described the dynamics of equine stomach development in foetal period and provided a solid background for further studies on histological structure of the gastric wall. A detailed development of the stomach wall and its structures in the prenatal life of a pig was described by Chrószcz [12]. Both works expanded our knowledge on stomach development in the foetal period and became good models for investigations subsequently carried out in horse foetuses. Moreover, a comparison of the prenatal development of the stomach in both above-mentioned species contributes not only to monogastric stomach equine and swine embryology, but to poligastric one ontogeny in ruminants.

## 2. Material and Methods

This study investigated 20 Wielkopolski horse breed foetuses from 4th to 11th month of gestation. The specimens were stored in the collection of the Division of Animal Anatomy (Wroclaw University of Environmental and Life Sciences). According to Polish law, tissue sample collection does not need an approval of an ethics committee if the study material is not derived from live organisms.

Foetus age was known (mare history of pregnancy, day of miscarriage) and verified using the crown-rump length (CRL) method [27,29,31,32]. Subsequently, the accessible material was divided using the population cross-sections method [12,13,14,15,33] into three age groups, five foetuses each (n = 5):

- the first age group—4th–5th month of gestation,
- the second age group—7th–8th month of gestation,
- the third age group—10th–11th month of gestation.

Additionally, the fourth group of five adult animals (5–8 years old) was used as a reference group to compare the fully developed stomach wall features with subsequent stages of histological structures formed in the foetal period.

All foetuses were preserved in 4% buffered paraformaldehyde solution for two to three months.

**Figure 1:**
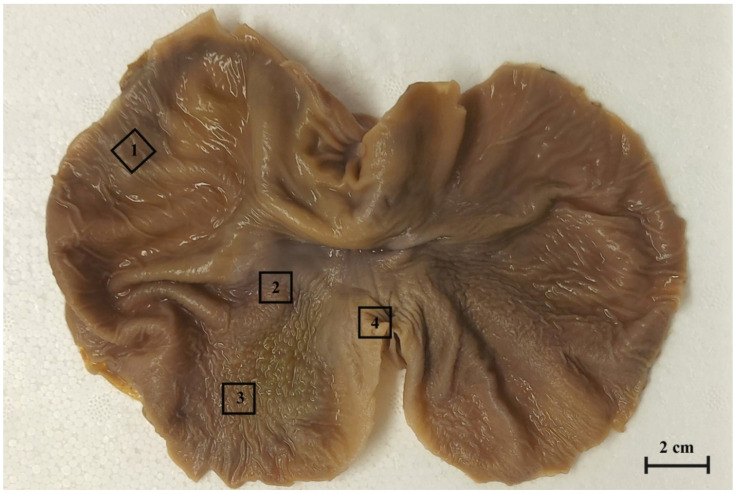
The gastric mucosa of equine foetal stomach (the 3rd age group) with the rectangular marks of sample collection areas. 1. the blind ventricular sac, 2. the plicated margin edge/cardiac part, 3. the body of stomach, 4. the pyloric part.

For a histological analysis, the tissue samples of the stomach wall (rectangular, 1 cm^2^ in size) were collected from selected regions of the stomach (blind ventricular sac, cardiac part/plicated edge margin, stomach body, and pyloric part of stomach (Figure 1) and fixed in 4% buffered paraformaldehyde for 48 h.

Next, the tissue samples were rinsed in running water for 24 h, and dehydrated with series concentrations of ethanol (50%, 70%, 96%, 100%) and isopropanol. Then, the stomach wall samples were impregnated with histological paraffin I (2 h) and paraffin II (24 h). Next, the material was embedded in paraffin wax and sectioned using a rotary microtome (Zeiss Hyrax M25; Carl Zeiss, Jena, Germany) into 7 μm sections. The histological slides were stained using a standard histological protocol (haematoxylin-eosin; Masson-Goldner, PAS) and mounted with cover slips.

The slides were observed in a light microscope (Zeiss Axio Scope A1; Carl Zeiss, Jena, Germany) equipped with software (Axiovision software, Carl Zeiss Microscopy, Jena, Germany) enabling morphometric and photographic documentation of the observed structures. The histometry of the stomach wall was carried out using an electronic tool calibrated for each magnification of the microscope. The following measurements were taken: nonglandular epithelium thickness, gastric glands depth, mucous membrane thickness, submucosa thickness, and muscular layer thickness. Each measurement was repeated three times and its mean value was computed.

The statistical analysis was carried out using OriginPro package, version 2021 (OriginLab Corporation, Northampton, MA, USA). All individual parameters were averaged, and standard deviation (SD) was calculated. Simple Fit application in OriginPro was used to present the regression curves. The results were plotted on linear and nonlinear graphs (allometric and polynomial approximation). As a measure of linear fit, where |r| = 1 means the strongest association of the two variables, Pearson’s correlation coefficient ® was used. As a measure of nonlinear curve fit, the correlation coefficient (R^2^ COD) was used, where R^2^ = 1 means that the fitted line explains all the variability in the response data around its mean. All results are presented in figures, tables, and diagrams.

## 3. Results

### 3.1. Microanatomy of the Stomach

**Figure 2:**
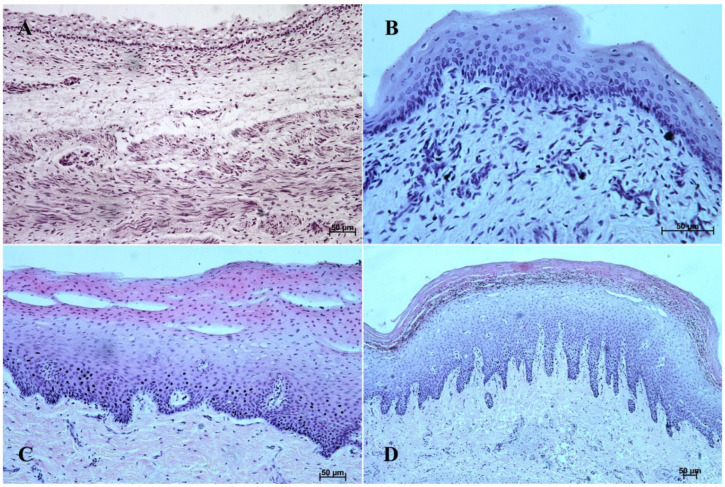
Histological structure of the stomach mucosa in the nonglandular part (hematoxylin-eosin staining). (**A**)—the first age group (20×); (**B**)—the second age group (40×); (**C**)—the third group (20×); (**D**)—the reference adult group (10×).

**Figure 3:**
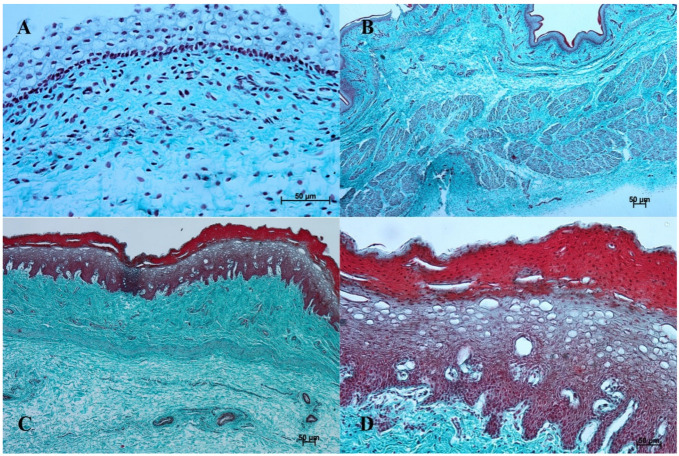
Histological structure of the stomach mucosa in the nonglandular part (Masson-Goldner staining). (**A**)—the first age group (20×); (**B**)—the second age group (40×); (**C**)—the third group (20×); (**D**)—the reference adult group (10×).

In the first age group, the wall of the stomach was divided into nonglandular and glandular part. Primordium of the blind ventricular sac was covered with a thin layer of multiple epithelium (Figure 2A and Figure 3A), and the glandular mucosa formed superficial concave areas, which were the beginning of the gastric pits. 

**Figure 4:**
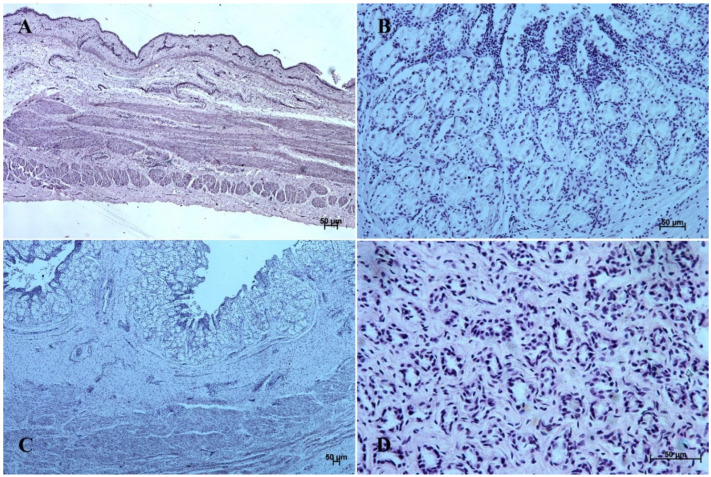
Histological structure of stomach mucosa in the cardiac part (hematoxylin-eosin staining). (**A**)—the first age group (10×); (**B**)—the second age group (20×); (**C**)—the third group (5×); (**D**)—the reference adult group (40×).

**Figure 5:**
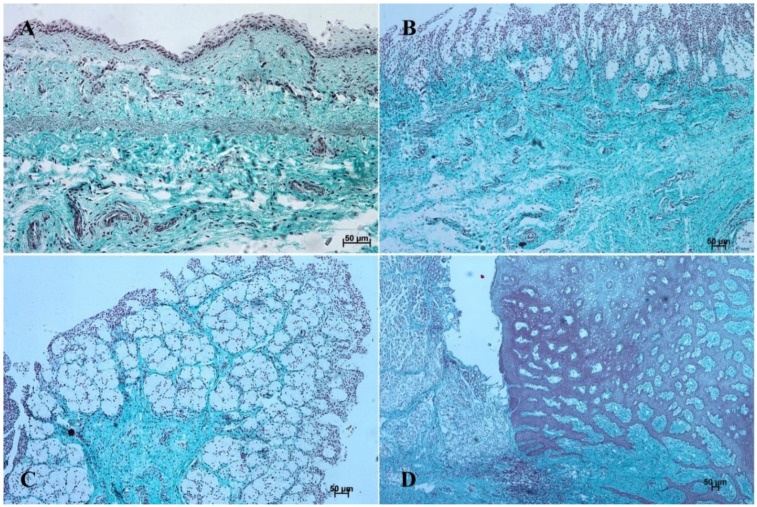
Histological structure of stomach mucosa in the cardiac part (Masson-Goldner staining). (**A**)—the first age group (20×); (**B**)—the second age group (10×); (**C**)—the third group (5×); (**D**)—the reference adult group (10×).

**Figure 6:**
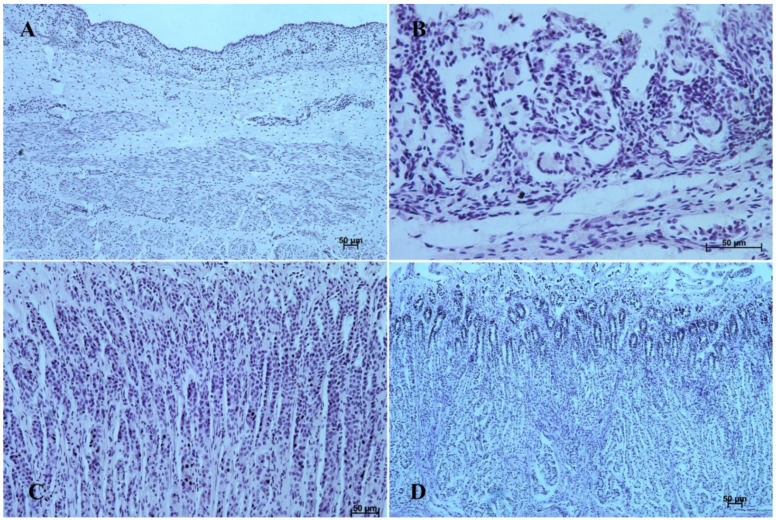
Histological structure of stomach mucosa in the pyloric part (hematoxylin-eosin staining). (**A**)—the first age group (20×); (**B**)—the second age group (10×); (**C**)—the third group (20×); (**D**)—the reference adult group (10×).

**Figure 7:**
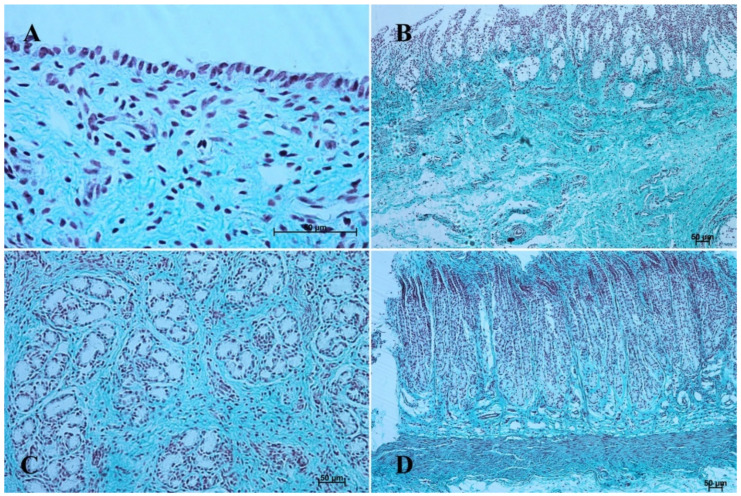
Histological structure of stomach mucosa in the pyloric part (Masson-Goldner staining). (**A**)—the first age group (63×); (**B**)—the second age group (10×); (**C**)—the third group (40×); (**D**) —the reference adult group (10×).

**Figure 8:**
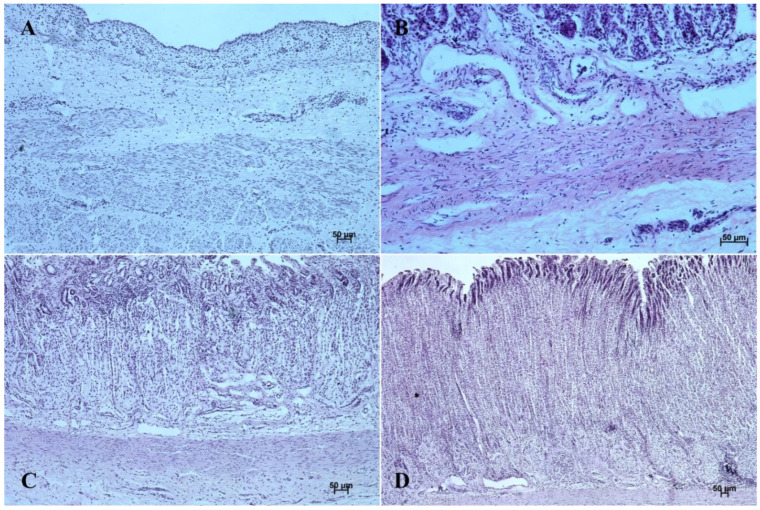
Histological structure of stomach mucosa in the fundic part (hematoxylin-eosin staining). (**A**)—the first age group (20×); (**B**)—the second age group (20×); (**C**)—the third group (10×); (**D**)—the reference adult group (5×).

**Figure 9:**
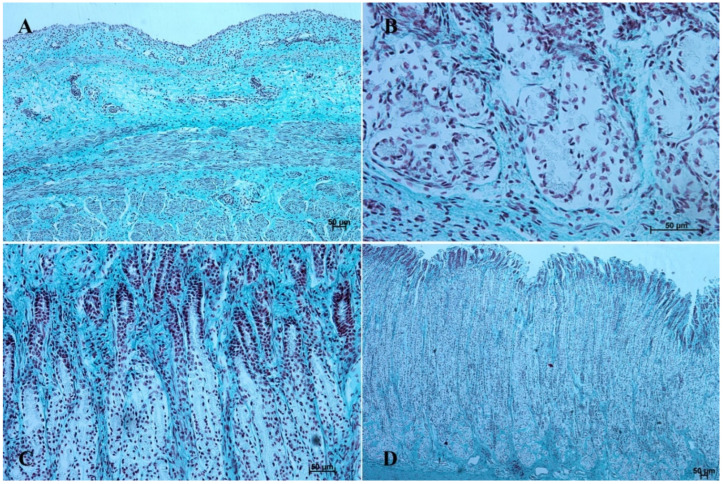
Histological structure of stomach mucosa in the fundic part (Masson-Goldner staining). (**A**)—the first age group (20×); (**B**)—the second age group (40×); (**C**)—the third group (20×); (**D**)—the reference adult group (5×).

The entire glandular mucosa was covered with a simple columnar epithelium (Figure 4A, Figure 5A, Figure 6A, Figure 7A, Figure 8A, and Figure 9A).

**Figure 10:**
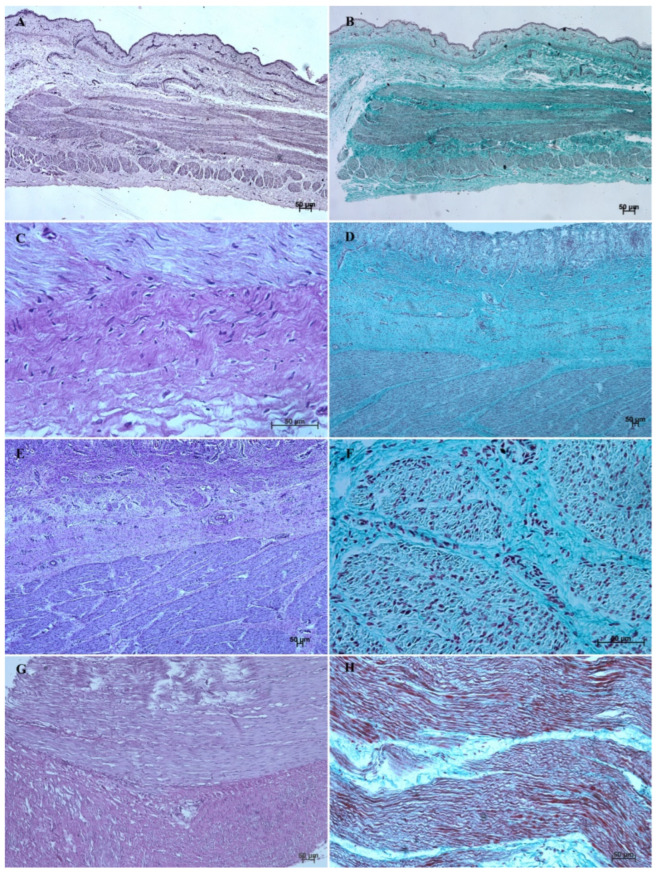
Histological structure of the muscular layer of stomach wall (hematoxylin-eosin staining and Masson-Goldner staining). (**A**,**B**)—the first age group (20×); (**C**,**D**)—the second age group (40× and 5×); (**E**,**F**)—the third age group (5× and 40×); (**G**,**H**)—the reference adult group (10× and 20×).

The *lamina propria mucosae* was formed by mesenchyme and a small amount of blood vessels. The muscular layer of the mucosa was absent (nonglandular and pyloric part of the stomach) or not well distinguishable (body of the stomach, the cardiac part). Thus, a clear division of the mucosa and submucosa was not possible (Figure 2A, Figure 4A, Figure 5A, Figure 6A, Figure 7B, Figure 8A,B and Figure 9A). The muscular layer of the stomach was divided into internal and external layer and was formed by myoblasts (Figure 10).

In the second age group, we could observe increasing thickness of the nonglandular multiple plane epithelium (Figure 2B and Figure 3B), and the primordium of plicated gastric edge was visible. The signs of epithelial cell desquamation were noted and the cylindrical cells of the germinative layer and basal membrane were discernible.

The glandular stomach develops into three distinguishable parts, which correspond to cardiac, fundic, and pyloric gland regions of the stomach wall (Figure 4B, Figure 5B, Figure 6B, Figure 7B, Figure 8B, and Figure 9B). The first parietal cells (*exocrinocytus parietalis*) were visible in the wall of the gland. Simple cylindrical epithelium with a basal membrane covered the *lamina propria mucosae* (Figure 9B).

The myocytes of the *lamina muscularis mucosae* separated the mucosa from the submucosa. Both layers were formed by loose connective tissue, with fibroblasts, collagen fibres, and capillary blood vessels (Figure 3B, Figure 5B, Figure 6B, Figure 7B, and Figure 8B). *Tunica muscularis* of the stomach was divided into an internal and external layer. Both of them were formed by fusiform myocytes (Figure 10). The glandular mucosa protruded into the connective tissue of mucosa forming the gastric glands. The cardiac glands were less deep than the fundic and pyloric ones.

In the third age group the gastric wall stratigraphy was typical of the mammalian stomach. The nonglandular part was covered with a thick layer of multiple, nonkeratinized plane epithelium (Figure 2C and Figure 3C).

The *lamina muscularis mucosae*, *submucosa* and both layers of *tunica muscularis* were visible (Figure 10). The gastric glands were developed and penetrated the connective stroma of the mucosa deep into its muscular layer forming large complexes of glandular tissue, especially in the fundic and pyloric part of the glandular mucosa (Figure 4C, Figure 5C, Figure 6C, Figure 7C, Figure 8C, and Figure 9C).

**Figure 11:**
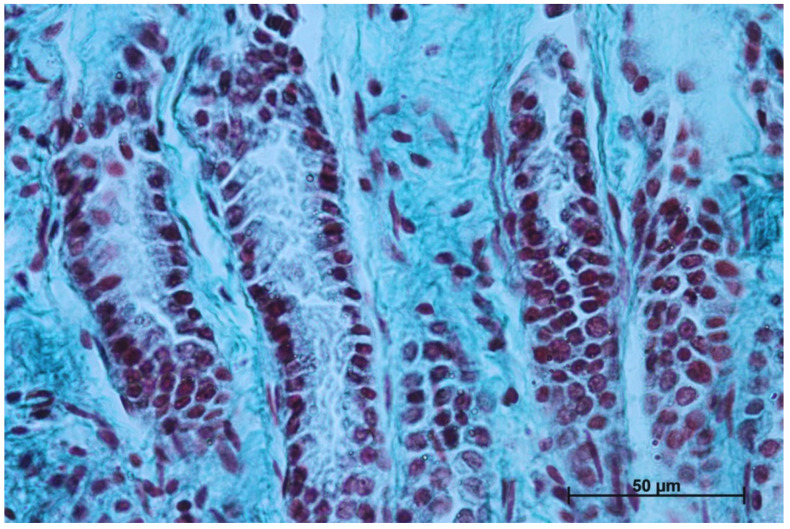
Body of the fundic glands with visible chief and parietal cells (Masson-Goldner staining, 63×).

The gastric glands can be classified as tubular glands with direct connection with the gastric pits. The chief cells (*exocrinocytus principalis*) and the parietal cells (*exocrinocitus parietalis*) were observed in developing fundic glands (Figure 11).

**Figure 12:**
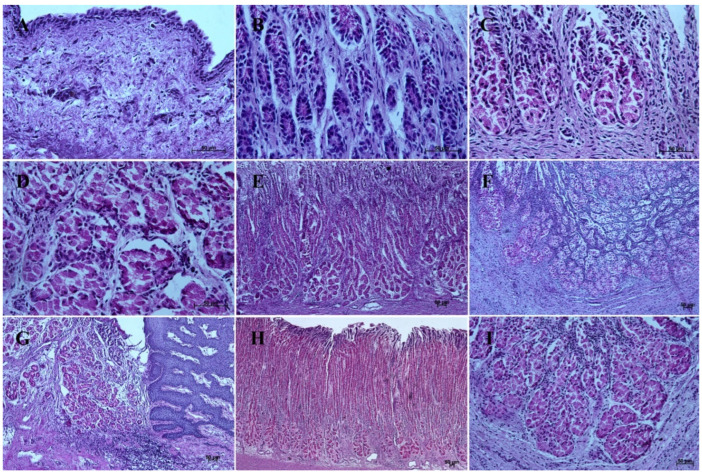
Histological structure of stomach mucosa in the glandular part (PAS staining). (**A**)—the first age group (40×); (**B**,**C**)—the beginning of mucus secretion by the gastric glands in the second age group (20× and 40×); (**D**)—the cardiac glands in the third age group (40×); (**E**)—the fundic glands in the third age group (20×); (**F**)—the pyloric glands in the third age group (40×); (**G**)—the cardiac glands in the reference adult group (10×); (**H**)—the fundic glands in the reference adult group (5×); (**I**)—the pyloric glands in the reference adult group (20×).

In contrast with the first age group, where the simple cylindrical epithelium began to protrude into the *lamina propria mucosae* and no specialized or histologically differentiated cells were observed, and the second age group, where the first parietal cells were visible, in the third age group both cell types were present. A comparison of the fundic glands in the second and third age group showed that the chief cells were evenly spread within the wall of the gastric glands, while the parietal cells were first identified in the middle section of developing gastric glands (the body of the gastric gland), and then spread superficially towards the isthmus of the gland and deeply towards the fundus of the gland. Moreover, in the third age group the mucous cells of the gland neck were visible in histological specimens (Figure 6C). The glandular mucosa showed no PAS-positive reaction (Figure 12A). The gastric glands of the second age group mucosa already began to produce a mucous secretion (moderate PAS-positive) (Figure 12B,C), while in the third age group the staining showed intense PAS-positive reaction in all glandular parts of the stomach mucosa (Figure 12D–F).

Regarding cardiac glands in the third age group (PAS staining, 40×), due to the volume of immunohistochemical results, detailed characterization of the fundic and pyloric glands with APUD cells identification will be the aim of a separate work.

The muscular layer of the stomach is built by myocytes that form subsequent layers. Especially strong development of the gastric muscular layer was observed in the pyloric part, together with strong accumulation of fibroblasts, collagen fibres, and blood vessels (Figure 10E,F).

The reference group (adult animals) presented a typical structure of the stomach wall, which was compared with the foetal stomach wall (Figure 2D, Figure 3D, Figure 4D, Figure 5D, Figure 6D, Figure 7D, Figure 8D and Figure 9D). Even though all basic structures were already developed in the third age group, some quantitative changes were detectable in comparison with the adult stomach. The majority of the changes included an increase in thickness of subsequent layers of the stomach wall and enlargement of the gastric gland complexes. The statistical analysis of histometric data provided further information. Moreover, the quantitative composition of the gastric wall layers and cells was similar to that observed in the third age group. PAS-positive glandular tissue reaction justified our final assumption that mucus production activity of the stomach mucosa in the third age group and postnatally is similar.

### 3.2. Histometry of the Stomach Wall

The microanatomical analysis included histometry of the stomach wall (Table 1, Table 2, Table 3 and Table 4).

We assessed the thickness of all basic layers (*mucosa*, *submucosa*, *tunica muscularis*) and their parts (epithelium/glands, *lamina propria mucosae*, *lamina muscularis mucosae*, *stratum internum* and *externum* of stomach muscular layer). The growth rate of subsequent layers was statistically analysed and compared with the foetus CRL (Table 5).

**Figure 13:**
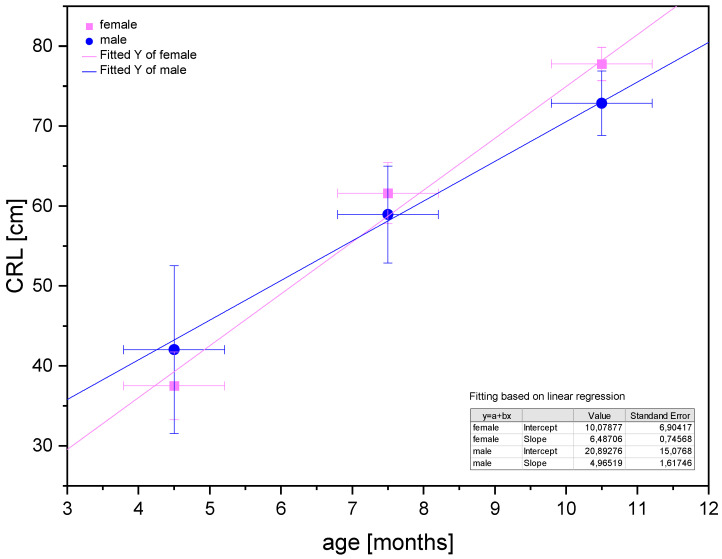
Linear regression of CRL values in the foetal period for male and female foetuses proving the isometric growth of the CRL (after Poradowski and Chrószcz [15]).

Poradowski and Chrószcz [15] proved an isometric growth of CRL in the foetal period between the 4th and 11th months of gestation (Figure 13).

The stomach wall structures were the subject of morphometric analysis in all four anatomical parts of the organ: the blind ventricular sac, the plicated margin edge/the cardiac part, the body of the stomach, and the pyloric part.

### 3.3. Blind Ventricular Sac

**Figure 14:**
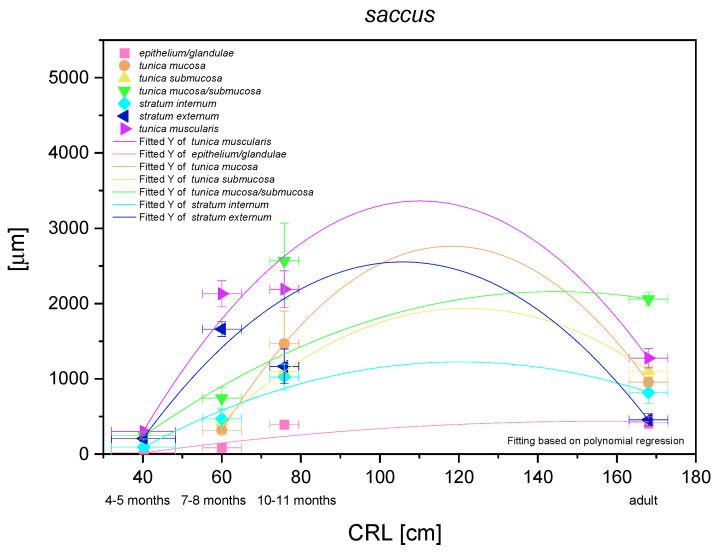
Histometric analysis of the stomach wall structures in the blind ventricular sac (saccus). The figure shows nonlinear regression curves of all metric parameters in all three age groups and adult reference group.

The results of statistical analysis of the results for blind ventricular sac are presented in Figure 14. All investigated parameters yielded nonlinear regression curves, which indicated the allometric growth rate in comparison with CRL. The multistratified plane epithelium of the nonglandular part of the stomach yielded a logarithmic regression curve (negative allometric growth) in the entire foetal period. It can be concluded that the thickness of the epithelium grew slower than of other parts of the stomach wall. It was relatively thin and the thickness of epithelium in the third age group and in the adult reference group was comparable. Taking under consideration the mucosa/submucosa (the lack of full separation of the layers in the first age group), the results proved a more significant and stronger negative allometric growth in the foetal period. A slight decrease of this parameter in the adult reference group can be explained by the stomach size differences between a new-born and an adult, where the mucosa/submucosa thickness did not take part in stretching the stomach wall postnatally. On the other hand, a comparison of separate growth of the mucosa and submucosa also showed negative allometric growth in the second and third age group, where the mucosa metric parameters were greater than those estimated for the submucosa. Therefore, after separation of the mucosa from the submucosa in the second age group, the significance of mucosa in the stomach growth was found greater than that of the submucosa in the mentioned stomach region. The greatest growth rate in the blind ventricular sac was observed for the muscular layer *in toto*. After separation of *stratum extremum* from *stratum internum*, the first one showed more intense growth rate, playing the most important role in the stomach wall thickness increase. All metric values in adult reference group were lower than those achieved prenatally. The greatest decrease was seen in the epithelium, *stratum internum*, and mucosa/submucosa thickness (Figure 3D).

### 3.4. Plicated Edge Margin/Cardiac Part

**Figure 15:**
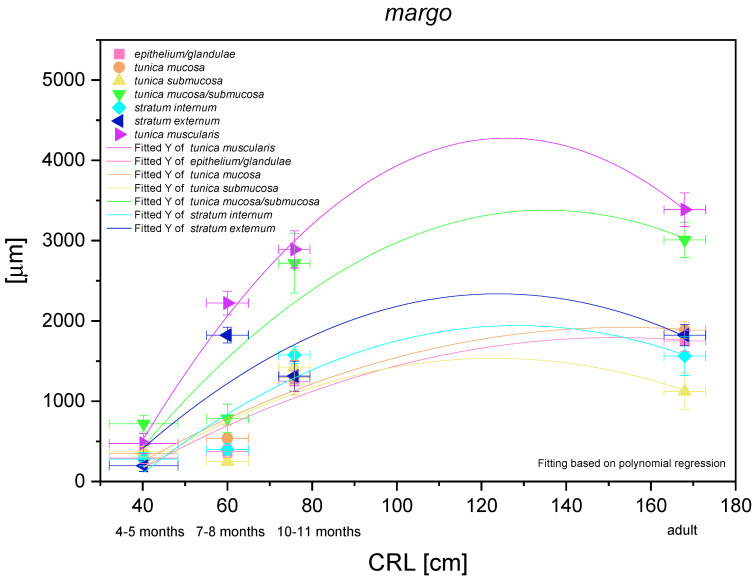
Histometric analysis of the stomach wall structures in the plicated edge margin/the cardiac part of the stomach (*margo plicatus*). The figure shows nonlinear regression curves of all metric parameters in all three age groups and the adult reference group.

The histometric analysis of this typical for equine stomach region is presented in Figure 15. A nonlinear regression of all parameters in comparison with CRL was visible, thus the negative allometric growth of subsequent structures of the stomach wall could be proved. The gastric glands and surrounding *propria mucosae* showed the lowest growing rate (negative allometric), comparable in intensity (close morphological and functional relations between the stomach mucosa and the gastric glands). However, this only involved the parameters that did not decrease in comparison with the adult reference group. The submucosa of this part of the stomach grew similarly to both above-mentioned parameters in the foetal period, and slightly decreased its participation in the stomach wall thickness in the adult reference group. Finally, the stomach muscular layer showed the greatest growth rate in the foetal period, but simultaneously the thickness of this layer was similar to that in the adult reference group. When we compared the *stratum externum* and *stratum internum* growth dynamics, we saw a moderately more intense growth of the *stratum externum*. Again, the values estimated in the third age group and in the adult reference group were comparable (with regard to CRL). In summary, the greatest role in stomach wall thickness increase can be ascribed to the mucosa/submucosa and the muscular layer *in toto*, but the crucial structures were the *stratum externum* and the mucosa with strong development of the cardiac glands. A similar assumption can be made based on the comparison of metric values of the mucosa (with the gastric glands) and the external stratum of the muscular layer in the adult reference group (Figure 5D and Figure 12G).

### 3.5. Body of the Stomach

**Figure 16:**
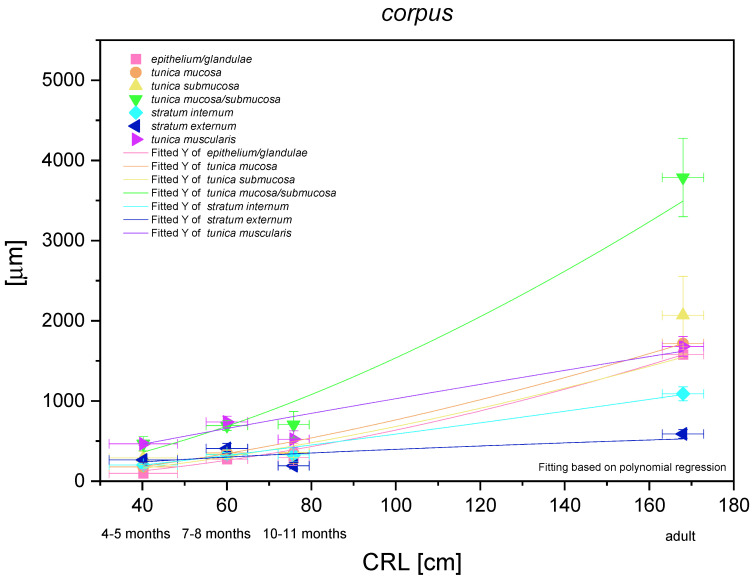
Histometric analysis of the stomach wall structures in the body of the stomach (corpus). The figure shows nonlinear and exponential regression curves of all metric parameters in all three age groups and the adult reference group.

The most functionally important part of the stomach (the fundic part) was analysed metrically and demonstrated a nonlinear and exponential growth rate (Figure 16). The mucosa/submucosa growth rate was the strongest (positive allometric) in the entire foetal period. Independent metric measurements of the mucosa (and the fundic glands) and the submucosa showed a similar intensity of positive allometric growth (in comparison with CRL). Growth of the stomach muscular layer *in toto* seemed to be near to linear in comparison with CRL, but the *stratum internum* played a more significant role in the stomach wall growth rate than the *stratum externum* (quasi linear regression curve). The fundic glands growth rate (positive allometric) proved the greatest role of the development of these glands in the stomach wall thickness prenatally and postnatally as compared with the cardiac and pyloric glands.

### 3.6. The Pyloric Part

**Figure 17:**
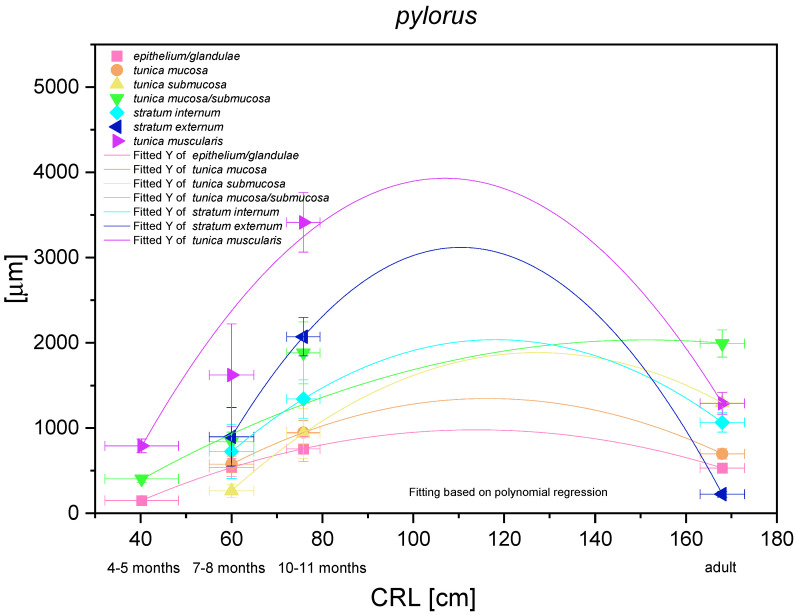
Histometric analysis of the stomach wall structures in the pyloric part (pylorus). The figure shows nonlinear and exponential regression curves of all metric parameters in all three age groups and the adult reference group.

The histometric analysis of the pyloric part of the stomach revealed a nonlinear regression (negative allometric growth) as compared with CRL (Figure 17). The mucosa/submucosa *in toto* (not separated in the first age group) showed allometric negative growth of low intensity, and the metric values in the third age group and the adult reference group were different (thickness of the mucosa/submucosa of the pyloric part increased postnatally). The fact that the mucosa and submucosa were separated in the second age group indicated that the submucosa growth rate was negatively allometric, but faster than that of the mucosa and its gland layer thickness growth rate. Similar to other glandular parts of the stomach mucosa, the growth rate of the pyloric glands and the mucosa thickness were comparable. The muscular layer of the pyloric part of the stomach showed very intense allometric growth in the foetal period. The *stratum externum* played much more important role in the increase of the muscular layer thickness. Simultaneously, a comparison between the third age group and the adult reference group revealed that the muscular layer underwent the strongest changes connected with stretching of the stomach wall postnatally, which were less visible in the mucosa and submucosa.

The CRL estimated in the foetuses was analysed together with the corresponding morphometric value estimated in the adult reference group. Except for the body of stomach histometry, in the blind ventricular sac, plicated edge margin/cardiac part, and the pyloric part of the stomach, the same pattern was seen. Most of the metric values decreased showing negative allometric growth in comparison with CRL. Only the multistratified plane epithelium of the blind ventricular sac and the mucosa/submucosa *in toto* reached the values that did not decrease in comparison with the adult reference group. The growth of the stomach body yielded an exponential regression curve in comparison with CRL, which described a more intense growth rate of the measured parameters within the most functionally important part of the organ.

## 4. Discussion

The stomach morphology in adult domestic mammals is well known and described in detail in the majority of veterinary anatomy textbooks [30,34]. The embryology of domestic mammals is aimed at the early phase of the prenatal development (the embryonic period), and more advanced stages of gestation are usually described only in general [9,10]. This tendency includes equine stomach morphology in the foetal period [28,29]. Detailed morphology and development of equine stomach in the foetal period was described by Poradowski and Chrószcz [15]. A similarly large anatomical and topographical study in domestic pig foetuses was carried out by Chrószcz [13] and it proved the usefulness of such an analysis as well as offered a valuable presentation of processes observed in more advanced phases of gestation. Similar to the investigations on morphology, embryology, and histometry of porcine gastric wall in the foetal period, this work elaborates on the development of accessible histological structures of equine stomach wall in the foetal period [12].

The equine stomach microanatomy showed signs of its division into the nonglandular and glandular part in the first age group. The primordium of blind ventricular sac mucosa covered with a thin layer of multiple layer epithelium partly resembled the histological picture of corresponding porcine mucosa at an early stage of the foetal period [12]. Even though in the nonglandular and pyloric parts of the stomach, the mucosa is not clearly separated from the submucosa, the developed muscular layer of the mucosa is visible in the plicated edge margin/the cardiac part and in the stomach body (Figure 4 and Figure 5). In our previous paper on swine, the porcine stomach mucosa was covered with a simple columnar epithelium, without any visible nonglandular epithelium between 35th and 40th day of gestation [12]. Contrary to that, Georgieva and Gerov [3] pointed out that the pig stomach mucosa epithelium was double layered epithelium covering the *propria mucosae*. These differences can be due to different methods of the foetal age estimation or to the fact that different parts of the stomach wall were investigated. In swine, the nonglandular part of the stomach mucosa surrounding the cardiac orifice is very narrow [30]. It is therefore possible that Chrószcz [12] described the glandular part and Georgieva and Gerov [3] aimed at less advanced stage of the mucosa development (30th day of gestation). In contrast with the above-mentioned findings in swine foetal stomach, the nonglandular mucosa of equine foetus stomach in the first age group was covered with a thin multiple layer epithelium (Figure 2A and Figure 3A), and the glandular mucosa formed superficial concave areas that represented early gastric pits and were covered with single cylindrical epithelium (Figure 4A, Figure 5A, Figure 6A, Figure 7A, Figure 8A and Figure 9A). Similar to the histological structure of porcine stomach in early foetal period (35th–40th day of gestation), the equine stomach body wall consisted of the *lamina propria mucosae* built of mesenchyme and small amount of blood vessels. The submucosa, separated or not from the mucosa, showed similar morphology. The muscular layer of the stomach was not divided into the external and internal layer, which corresponded with the histological picture observed in the pig between day 35 and 40 day of gestation [12]. The gastric pits visible in the first age group corresponded with similar structures spotted during the mucosa transformation on day 56 of gestation in swine stomach [12]. Jackowiak and Godynicki [35] linked the formation of gastric pits with the development of vascular architecture of prenatal stomach in horses. Therefore, in the second and third age group the blood vessels were more numerous.

The second age group showed a more advanced stage of gastric wall development involving increasing thickness of the nonglandular multiple plane epithelium (Figure 2B and Figure 3B) and the plicated edge margin formation (Figure 3B). The histological picture of swine foetal stomach confirmed the existence of nonglandular part of the mucosa on 56th day of gestation, but a significant increase of its thickness was seen on day 82 [12]. This can probably be explained by the length of gestation in both species (114 days in pigs and 330 days in horses).

The nonglandular epithelium development occurred at similar stages of pregnancy in both species. Considering the glandular part of the stomach mucosa development, separated cardiac, fundic, and pyloric parts of the stomach mucosa were visible in the second age group (Figure 4B, Figure 5B, Figure 6B, Figure 7B, Figure 8B and Figure 9B). Similar division of the glandular mucosa in swine foetal stomach was seen on day 60 of gestation [12]. The parietal cells were detectable (Figure 11), and their earlier differentiation than of the chief cells was also observed in swine [3,12]. The parietal cells of swine foetal gastric mucosa were present on day 60 of gestation, while the first chief cells appeared on day 107 [12]. Georgieva and Gerov [3] reported the presence of the chief cells in pig stomach on day 90 of gestation, similarly as Rüsse and Sinowatz [10]. The first PAS-positive reaction within the gastric glands was observed at the same time [3]. A moderately positive PAS staining was visible in the second age group (Figure 12B,C), and intense PAS-positive reaction was seen in the third age group (Figure 12D–F). This proved that the excretory activity of the stomach mucous glands begins with the parietal cell differentiation both in porcine and equine prenatal period. It seems reasonable, as the function of the stomach mucosa is to defend the organ against digestive and degenerative activity of the parietal cell secretion. Differentiation of the chief cells within the fundic gland wall took place later, therefore the increase in gastric mucosa mucus production increases as the birth approaches. This process is reflected in the stomach gland allometric negative growth in the cardiac and pyloric part, and positive allometric growth of the stomach body glandular tissue (Figure 15, Figure 16 and Figure 17). The fundic gland cells, similarly as the chief cells, spread evenly within the gland wall, while the parietal cells were primary identified in the wall of the gastric gland body, from where they spread in both directions (toward the isthmus and the fundus of the gland). The parietal cells had untypical pyramidal shape and they were located in groups among the epithelial cells. The first parietal cells in swine fundic glands were seen at the base of deeper gastric pits [12]. Our findings were similar to those of other authors [3,8,36].

Common presence of the parietal cells in pairs proved their origin from one precursor cell [37]. The parietal cells were visible in superficial parts of the gastric glands on day 60 of swine pregnancy [12]. All gastric pit cell types, i.e., surface mucous cells (*epitheliocyti superficiales*), neck cells (*mucocyti cervicales*), and parietal (oxyntic) cells (*exocrinocyti parietales*), come directly from the stem cells, while the chief (zymogenic) cells (*exocrinocyti principales*) come from the neck cells [18,19,20,21,22,23,38]. The presence of the chief cells and the parietal cells within the wall of the gastric gland in equine foetus probably indicated a more advanced stage of glandular cells differentiation than that described in swine. A detailed description of the gastric glands, together with APUD cells, will be the aim of a separate work. The *lamina muscularis mucosae* separating the mucosa from the submucosa is built by myocytes. Both layers of the gastric wall consist of loose connective tissue with fibroblasts, collagen fibres, and capillary blood vessels (Figure 8B).

Finally, the stomach muscular layer is divided into internal and external layers, built by fusiform myocytes (Figure 10). The histological structure of all described layers of the stomach wall resembled that observed in swine foetuses at more advanced stages of the foetal period (82nd to 107th day of gestation) [12].

In the third age group, all layers of the stomach wall were distinguishable and developed comparably to the swine foetal gastric wall at late stages of gestation [12]. The nonglandular epithelium was formed with a thick layer of multiple nonkeratinized plane epithelium (Figure 2D and Figure 3D). Similar histology was observed in swine foetuses in proventricular part of the stomach [12]. The groups of tubular complex glands deeply penetrating the muscular layer of the gastric mucosa were visible in all three parts of glandular mucosa of the stomach wall (Figure 4D, Figure 5D, Figure 6D, Figure 7D, Figure 8D, and Figure 9D). The chief and parietal cells were observed in the fundic glands of the stomach mucosa (Figure 11). Moreover, the mucous cells of the neck of the glands were visible (Figure 6C). The gastric glands in pig foetuses on day 114 of gestation (terminal stage of pregnancy) showed a similar structure [12]. Therefore, it may be concluded that the basic structure of glandular mucosa develops prenatally. The myocytes formed both the muscular layer of the mucosa and the muscular layer of the stomach. The latter was especially well developed in the pyloric region (Figure 10F). Similar observations concerning the muscular layer of the pylorus were made in pig foetuses [12]. Bal and Ghoshal [39] pointed out that the circular layer of muscular tissue was the most developed in the pyloric part of the stomach. Postnatally, the strongest part of the gastric muscular layer is the musculature of the pylorus that serves as the anatomical border between the acidic and basic environments of the stomach and the duodenum, and as a suction and pressure pump crucial for the peristalsis of the alimentary tract.

It is well known that the development of alimentary tract does not finish at the moment of birth. The quantitative and qualitative changes occur also postnatally [3,12,16,40]. The adult reference group showed a typical histological structure of the stomach wall comparable to that of the foetal stomach wall status (Figure 2C, Figure 3C, Figure 4C, Figure 5C, Figure 6C, Figure 7C, Figure 8C, and Figure 9C). The majority of morphological changes consisted in the increase in the dimensions of subsequent layers of the stomach (Figure 2D, Figure 3D, Figure 4D, Figure 5D, Figure 6D, Figure 7D, Figure 8D, and Figure 9D). Even though this study discusses similarities and differences of a complex monogastric stomach of swine and intermediate in equine foetuses, a polygastric stomach of ruminants shows a similar cellular structure [41]. The structure of a stomach in rabbits established on days 22–30 of gestation was similar to that observed in adult animals [35]. Contrary to that, studies carried in domestic cats proved an intense development of the fundic and pyloric glands until the age of eight months postnatally [8]. Studies on the functions of gastric glands in rats indicated that animal diet significantly affects the stomach mucosa maturation [42] (Kammeraad, 1942). Finally, Xu et al. [43] proved that the strongest growth rate of the stomach wall was observed in the body of the stomach and the fundic glands, thus was visible especially within the gastric mucosa. All these remarks clearly demonstrate that the stomach wall development is not finished on the day of birth. The morphology of the stomach wall in the third and reference age group is similar. The distribution of chief and parietal cells within the gland wall was similar in adult gastric glands. The glandular mucosa showed intense PAS-positive reaction both in the third age group and reference group. The differences consisted in the values of metric parameters, which were lower in the adult reference group than in the third age group and especially visible in the epithelium, *stratum internum*, and mucosa/submucosa thickness. The positive allometric growth rate of the fundic glands located within the mucosa and external muscular layer played the most important role in the stomach wall thickness increase and the organ adaptation to its function postnatally. The postnatal development of the stomach causes the stretching of the wall due to the increase in stomach’s size. This process was more visible in the muscular layer than in the mucosa and submucosa thickness decrease. The multistratified plane epithelium of the blind ventricular sac thickness was the only value which did not decrease postnatally.

Moreover, the development of gastric glands took place in perinatal and postnatal period, when they underwent dynamic changes influencing the stomach wall physiology and morphology [44,45,46]. The immunological system can affect the physiology of the pyloric mucosa exposed to pathogens and feeding methods [47]. This is not linked to the prenatal development of stomach but is important in adult animals.

The statistical analysis of histometric data is very important for the description of prenatal development of stomach in foetal period. In a huge anatomical study on stomach development in the foetal period Poradowski and Chrószcz [15] showed positive allometric growth for all investigated morphometric parameters and isometric growth of CRL. The differences in growth rate of basic parameters of the stomach morphometry made us assume that the stomach shape can be described as slightly bended and medium wide (the first age group) and sharply bended and wide with considerably sized blind ventricular sac (left) and strong pyloric part (right) in the second and third age group. A clear border between the nonglandular and glandular mucosa was visible as developing plicated edge margin in the second age group. Moderate changes in the stomach morphology in foetal period were also reported in domestic pig foetuses [13].

The histometry of the stomach wall in the blind ventricular sac, the plicated edge margin, and the pyloric part (Figure 14, Figure 15, Figure 16 and Figure 17) proved that all investigated parameters showed negative allometric growth as compared with linear growth of CRL and the foetus age. The multistratified plane epithelium thickness increased more slowly than that of the epithelium in other parts of the stomach and was comparable in the third age group and the adult reference group. The glandular mucosa thickness is strongly linked with the gastric gland development. The mucosa of the plicated edge margin and cardiac part of the stomach showed the most negative allometric growth and did not decrease in the adult reference group. Similar to the above-mentioned parts of the stomach mucosa, the growth of the pyloric glands and mucosa thickness was comparable and negative allometric. The fundic glands development differed showing positive allometric growth, which can be explained by the most intense increase in the fundic glands growth in developing stomach wall prenatally and postnatally as compared with the cardiac and pyloric glands. Our study carried out in pig foetuses showed positive allometric growth of the glandular mucosa from day 60 of gestation, and negative allometric growth was observed at the earliest stage of foetal period [12]. These differences are probably caused by the final organ size. The equine stomach is smaller than the porcine one in relation to the animal body size [30]. Therefore, the swine gastric mucosa grows quicker to be able to cover the entire surface of rapidly and intensively increasing size and volume of the stomach.

The mucosa and submucosa were not fully separated in the blind ventricular sac and the pyloric part of the stomach in the first age group. Therefore, all four parts of the histometric investigation included the mucosa/submucosa thickness (Figure 14, Figure 15, Figure 16 and Figure 17). The mucosa/submucosa of the blind ventricular sac showed significant decrease in the growth rate (negative allometric) in the foetal period as compared with the mucosa alone. The thickness of mucosa/submucosa in the adult reference group slightly decreased too, which can be due to the differences in the stomach size between a new-born and an adult. It seems that the mucosa/submucosa did not participate in the stomach wall expansion and stretching compensation. A comparison of the growth rate of the mucosa and submucosa independently clearly showed that negative allometric growth in the second and third age groups was more intense in the case of the mucosa than the submucosa, and the mentioned mucosa thickness increase played a much more important role in the stomach wall development in the advanced phase of the foetal period. In the plicated edge margin/the cardiac part of the stomach, the relations between the submucosa and the mucosa were similar. The morphology of the mucosa/submucosa layer in the pyloric part of the stomach resembled that observed in the blind ventricular sac (lack of full separation of the layers). Even though prenatally the thickness of this stomach wall layer grew in slightly negative allometric manner, a comparison of the third age group and the adult reference group showed different metric values. Moreover, when analysing both layers separately in the second age group we found that the submucosa growth rate was greater than that of the mucosa. The thickness of mucosa and its glandular tissue increased comparably. Finally, the growth rate of the mucosa/submucosa within the stomach body was strongly positive allometric throughout the entire foetal period. This corresponded with positive allometric growth rate of the most physiologically important gastric glands (the fundic glands). The mucosa growth in the pig foetal period also showed a positive allometric rate [12]. This made us conclude that positive allometric growth is typical for the body of the stomach in both species.

The muscular layer of the stomach wall is the most important structure in the context of the stomach peristaltic activity, stomach ability to increase in volume during food storage, and its function as the pyloric suction and pressure pump, which separates acidic and basic chemical environments of the stomach and duodenum lumen and is responsible for gradual release of pre-digested food from the stomach into the intestine [47,48]. In the blind ventricular sac muscular layer *in toto* showed significant growth rate and the *stratum externum* seemed to be the most important for the stomach wall increase (Figure 14). The muscular layer of the plicated edge margin/the cardiac part of the stomach showed the most intense growth rate in the foetal period. Simultaneously, the parameter in the perinatal period and in adult animals was comparable (Figure 15). Again, the growing rate of the *stratum internum* was more moderate than of the *stratum externum* and comparable in the third age group and the adult reference group. The growth of the muscular layer of the pyloric part of the stomach was strongly allometric in the foetal period. The *stratum externum* played a much more important role in the increase of the muscular layer thickness. A comparison of the third age group with the adult reference group demonstrated that the muscular layer underwent the greatest changes associated with stretching of the stomach wall postnatally, and the changes were less visible in the mucosa and submucosa. Considering the muscular layer of the stomach body, it may be stated that this layer *in toto* showed an almost linear growth rate (as compared with CRL). Moreover, in contrast with all earlier mentioned stomach wall parts, the *stratum internum* played a more important role in the stomach wall development than the *stratum externum*. This can be explained by the anatomical structure of the muscular layer, which is especially strong in the pyloric part, weaker in the blind ventricular sac or the plicated edge margin/the cardiac part of stomach, and the weakest in the stomach body. The *stratum externum* is located along the gastric curvatures, less prominent in the parietal and visceral surface of the stomach [30].

## 5. Conclusions

In summary, the most evident increase in thickness can be observed in the mucosa/submucosa and the muscular layer *in toto*. However, analysing them separately and together with the mucosa, we found that the growth of the mucosa with gastric glands and the *stratum externum* of the muscular layer is the most important for stomach wall compensation of the organ growth.

The values of the metric parameters estimated in all age groups and the adult reference group (with regard to CRL) were comparable, except for the body of the stomach wall. Negative allometric growth in the blind ventricular sac, the plicated edge margin/the cardiac part, and the pyloric part of the stomach did not correlate with positive allometric growth observed in the stomach body. In the swine foetal period, the mucosa showed positive allometric growth, the submucosa demonstrated allomeric negative growth, and the muscular layer growth rate can be described as the most constant and moderately positive allometric. The differences between pig and horse stomach wall development dynamics can be explained by the stomach volume intake in both species. The equine stomach is small and the swine stomach is large in comparison with the whole animal body size.

The monogastric complex stomach development in the foetal period seems to be a good example of equine and porcine comparative embryology, which is rarely a subject of more detailed studies. Even though in this paper we utilized anatomical and embryological sources, it seems important to stress their value for further studies. Classical morphological and morphometrical analyses are underappreciated. This work tried to prove that by exploring this field we may be able to explain the processes taking place during ontogeny. Moreover, there are open possibilities for further studies on a polygastric stomach of ruminants and a simple monogastric stomach of carnivores. Veterinary embryology focuses on the embryonic period, but the foetal period of organogenesis, even if less spectacular, also needs our attention.

## Figures and Tables

**Table 1:** Histometry of equine stomach—the pyloric part of the stomach (*pars pylorica*).

**Age [Months]**	**4–5**										
**Stomach Wall Layer [µm]**											**mean/sd**
*Epithelium*/*glandulae*	110.31	174.73	177.38	145.35	183.28	109.34	146.23	142.48	179.3	138.23	150.66 ± 27.45
*Tunica mucosa*	-	-	-	-	-	-	-	-	-	-	- -
*Tunica submucosa*	-	-	-	-	-	-	-	-	-	-	- -
*Tunica mucosa*/*submucosa*	375.75	331.89	484.57	421.34	389.78	402.37	388.67	456.85	402.1	378.28	403.2 ± 43.05
*Tunica muscularis*	744.64	825.25	663.22	734.23	893.21	804.4	793.78	699.3	837.12	909.53	790.47 ± 80.34
*Stratum internum*	-	-	-	-	-	-	-	-	-	-	- -
*Stratum externum*	-	-	-	-	-	-	-	-	-	-	- -

**Age [Months]**	**7–8**										
**Stomach Wall Layer [µm]**											**mean/sd**
*Epithelium*/*glandulae*	466.09	597.74	409.68	688.56	629.34	503.7	640.72	601.97	429.32	408.45	537.56 ± 105.74
*Tunica mucosa*	616.03	651.47	536.87	492.56	695.87	679.44	473.32	451.52	661.91	497.64	575.66 ± 94.53
*Tunica submucosa*	313.72	289.36	188.07	366.55	298.85	182.7	177.41	387.64	229.27	199.86	263.34 ± 78.4
*Tunica mucosa*/*submucosa*	929.8	940.8	724.9	859.1	994.7	862.1	650.7	839.2	891.2	697.5	839 ± 113.04
*Tunica muscularis*	1183.48	931.23	150.67	803.45	687.32	847.87	407.35	412.98	790.42	1024.84	723.96 ± 315.82
*Stratum internum*	1056.36	1219.87	111.83	906.78	614.03	1267.56	749.84	1167.22	897.13	982.57	897.31 ± 344.09
*Stratum externum*	2239.84	2151.1	262.5	1710.23	1301.35	2115.43	1157.19	1580.2	1687.55	2007.41	1621.28 ± 599.5

**Age [Months]**	**10–11**										
**Stomach Wall Layer [µm]**											**mean/sd**
*Epithelium*/*glandulae*	555.69	988.7	634.45	748.42	856.24	589.67	767.34	958.9	753.56	699.3	755.23 ± 145.8
*Tunica mucosa*	652.94	1067.23	855.48	1013.56	1104.8	953.34	863.21	879.47	1103.85	977.99	947.18 ± 139.77
*Tunica submucosa*	676.69	1614.4	559.38	824.78	757.2	948.78	1045.98	1106.34	867.93	945.2	934.66 ± 290.52
*Tunica mucosa*/*submucosa*	1329.63	2681.63	1414.86	1838.34	1862	1902.12	1909.19	1985.81	1971.78	1923.19	1881.9 ± 363.12
*Tunica muscularis*	1130.89	1023.32	1586.46	1568.34	1636.86	1467.24	1382.61	1089.33	1169.47	1353.76	1340.83 ± 224.88
*Stratum internum*	2283.9	1676.78	2272.15	2368.21	1987.04	1842.56	1992.1	2297.79	2005.32	1985.81	2071.16 ± 225.58
*Stratum externum*	3414.79	2700.1	3858.61	3936.55	3623.9	3309.8	3374.71	3387.12	3174.79	3339.57	3411.99 ± 349.65

**Age [Months]**	**Adult**										
**Stomach Wall Layer [µm]**											**mean/sd**
*Epithelium*/*glandulae*	480.01	501.4	529.96	595.67	565.32	530.02	546.34	583.78	466.11	513.97	531.26 ± 42.63
*Tunica mucosa*	655.62	651.23	706.21	663.57	765.31	771.83	700.26	613.1	657.83	789.82	697.47 ± 60.07
*Tunica submucosa*	1158.12	1089.49	1388.15	1435.73	1286.48	1389.65	1462.57	1173.78	1282.63	1271.74	1293.83 ± 125.58
*Tunica mucosa*/*submucosa*	1813.74	1740.72	2094.36	2099.3	2051.79	2161.48	2162.83	1786.88	1940.46	2061.56	1991.31 ± 159.15
*Tunica muscularis*	1060.29	1030.17	1012.31	997.46	1183.83	1098.34	1163.27	945.28	899.43	1267.45	1065.78 ± 113.62
*Stratum internum*	222.89	240.14	230.1	158.52	215.33	182.93	292.42	228.9	229.37	235.28	223.58 ± 35.29
*Stratum externum*	1283.18	1270.31	1242.41	1155.98	1399.16	1281.27	1455.69	1174.18	1128.8	1502.73	1289.37 ± 126.82

**Table 2:** Histometry of equine stomach—the stomach body (*corpus ventriculi*).

**Age [Months]**	**4–5**										
**Stomach Wall Layer [µm]**											**mean/sd**
*Epithelium*/*glandulae*	70.13	133.95	89.18	122.45	48.23	181.17	45.67	154.89	98.38	43.48	98.75 ± 48.4
*Tunica mucosa*	118.84	174.04	214.27	263.28	107.94	127.67	205.43	199.36	175.16	193.58	177.96 ± 48.31
*Tunica submucosa*	180.38	373.18	203.64	241.78	275.23	395.07	188.38	360.54	358.18	318.71	289.51 ± 82.16
*Tunica mucosa*/*submucosa*	299.22	547.22	417.91	505.06	383.17	522.74	393.81	559.9	533.34	512.29	467.47 ± 87.57
*Tunica muscularis*	203.55	222.2	189.61	181.1	198.82	230.85	177.68	178.31	221.43	216.37	201.99 ± 19.94
*Stratum internum*	268.59	240.05	275.75	261.34	219.73	259.71	297.84	285.74	284.05	262.28	265.51 ± 22.9
*Stratum externum*	472.14	462.25	465.36	442.44	418.55	490.56	475.52	464.05	505.48	478.65	467.5 ± 24.16

**Age [Months]**	**7–8**										
**Stomach Wall Layer [µm]**											**mean/sd**
*Epithelium*/*glandulae*	286.43	266.53	291.82	207.34	277.23	281.74	293.66	278.38	291.19	279.27	275.36 ± 25.28
*Tunica mucosa*	385.81	370.08	340.06	326.87	376.37	333.38	330.02	391.38	373.88	319.48	354.73 ± 27.24
*Tunica submucosa*	367.09	262.01	393.99	280.11	303.17	391.86	390.84	395.15	313.43	287.65	338.53 ± 54.18
*Tunica mucosa*/*submucosa*	752.9	632.09	734.05	606.98	679.54	725.24	720.86	786.53	687.31	607.13	693.26 ± 61.94
*Tunica muscularis*	306.94	320.74	350.31	316.49	304.6	350.65	318.89	321.6	323.82	390.91	330.5 ± 26.3
*Stratum internum*	422.22	337.23	433.47	482.23	348.73	495.34	477.95	387.21	321.88	380.48	408.67 ± 63.25
*Stratum externum*	729.16	657.97	783.78	798.72	653.33	845.99	796.84	708.81	645.7	771.39	739.169 ± 70.74

**Age [Months]**	**10–11**										
**Stomach Wall Layer [µm]**											**mean/sd**
*Epithelium*/*glandulae*	179.01	411.27	178.65	307.8	270.17	413.96	351.79	158.97	297.5	365.86	293.5 ± 95.55
*Tunica mucosa*	294.85	502.38	259.66	460.99	238.75	209.93	263.88	395.62	569.84	275.69	347.16 ± 125.61
*Tunica submucosa*	370.46	287.24	313.19	421.99	372.21	252.54	335.6	413.17	441.85	365.25	357.35 ± 60.75
*Tunica mucosa*/*submucosa*	665.31	789.62	572.85	882.98	610.96	462.47	599.48	808.79	1011.69	640.94	704.51 ± 165.2
*Tunica muscularis*	295.66	364.89	380.04	388.74	434.28	236.26	204.67	259.96	457.2	270.76	329.25 ± 87.12
*Stratum internum*	232.46	156.19	161.16	174.2	236.5	137.42	164.74	189.67	230.5	252.86	193.57 ± 40.9
*Stratum externum*	528.12	521.08	541.2	562.94	670.78	373.68	369.41	449.63	687.7	523.62	522.82 ± 106.56

**Age [Months]**	**Adult**										
**Stomach Wall Layer [µm]**											**mean/sd**
*Epithelium*/*glandulae*	1605.7	1529.65	1543.6	1689.37	1639.63	1611.64	1533.99	1606.52	1535.66	1496.09	1579.19 ± 60.42
*Tunica mucosa*	1777.4	1655.65	1673.96	1731.22	1699.6	1682.05	1735.84	1697.85	1771.52	1746.38	1717.15 ± 41.56
*Tunica submucosa*	2650.59	2496.4	1481.03	2065.56	2523.31	1436.85	2297.71	2425.52	1907.09	1415.29	2069.94 ± 483.69
*Tunica mucosa*/*submucosa*	4427.99	4152.05	3154.99	3796.78	4222.91	3118.9	4033.55	4123.37	3678.61	3161.67	3787.08 ± 489.53
*Tunica muscularis*	1181.19	1174.25	1143.01	954.74	995.36	1169.6	1014.76	1104.73	1153.04	1008.87	1089.96 ± 87.08
*Stratum internum*	579.29	613.72	558.95	599.13	532.83	689.1	539.54	568.61	671.15	545.25	589.76 ± 54.17
*Stratum externum*	1760.48	1787.97	1701.96	1553.87	1528.19	1858.7	1554.3	1673.34	1824.19	1554.12	1679.71 ± 125.6

**Table 3:** Histometry of equine stomach—the plicated edge margin (*margo plicatus*).

**Age [Months]**	**4–5**										
**Stomach Wall Layer [µm]**											**mean/sd**
*Epithelium*/*glandulae*	225.12	242.85	322.62	304.53	375.8	374.44	214.17	274.64	369.61	217.66	292.14 ± 66.42
*Tunica mucosa*	371.47	312.32	411.78	330.01	378.77	390.9	378.98	277.16	305.22	331.42	348.8 ± 43.56
*Tunica submucosa*	350.07	304.08	444.45	354.98	444.51	361.59	491.23	315.97	312.69	332.56	371.21 ± 65.34
*Tunica mucosa*/*submucosa*	721.54	616.4	856.23	684.99	823.28	752.49	870.21	593.13	617.91	663.98	720.02 ± 102.45
*Tunica muscularis*	356.24	351.24	276.23	205.61	120.44	393.72	235.58	251.1	348.27	241.83	278.03 ± 84.14
*Stratum internum*	210.37	106.52	190.48	204.21	211.44	362.74	112.56	178.11	185.84	203.28	196.56 ± 69.75
*Stratum externum*	566.61	457.76	466.71	409.82	331.88	756.46	348.14	429.21	534.11	445.11	474.58 ± 122.6

**Age [Months]**	**7–8**										
**Stomach Wall Layer [µm]**											**mean/sd**
*Epithelium*/*glandulae*	461.74	306.29	297.58	254.43	422.24	471.66	443.89	411.18	330.4	348.73	374.81 ± 76.8
*Tunica mucosa*	703.65	394.3	452.31	525.68	771.92	273.12	534.51	774.72	437.33	515.16	538.27 ± 165.72
*Tunica submucosa*	296.02	276.95	239.84	275.57	298.18	248.15	229.91	211.16	200.75	204.96	248.15 ± 36.91
*Tunica mucosa*/*submucosa*	999.67	671.25	692.15	801.25	1070.1	521.27	764.42	985.88	638.08	720.12	786.42 ± 178.04
*Tunica muscularis*	466.81	377.27	498.47	441.52	303.43	303.5	321.63	422.28	446.89	419.34	400.11 ± 70.12
*Stratum internum*	1973.09	1786.13	1843.43	1845.35	1761.84	1779.26	1732.9	1663.32	1871.7	1967.04	1822.41 ± 98.43
*Stratum externum*	2439.9	2163.4	2341.9	2286.87	2065.27	2082.76	2054.53	2085.6	2318.59	2386.38	2222.52 ± 147.74

**Age [Months]**	**10–11**										
**Stomach Wall Layer [µm]**											**mean/sd**
*Epithelium*/*glandulae*	1345.29	1435.26	1060.12	1305.17	1045.34	1283.62	1377.95	1246.98	1178.15	1212.11	1249 ± 128.45
*Tunica mucosa*	1617.27	1175.5	1532.5	1045.7	1156.45	1369.56	1030.08	1144.94	1338.05	1537.34	1294.74 ± 214.67
*Tunica submucosa*	1790.8	1258.61	1705.89	1504.97	1333.89	1551.13	1274.1	1256.57	1408.58	1143.4	1422.79 ± 211.26
*Tunica mucosa*/*submucosa*	3408.07	2434.11	3238.39	2550.67	2490.34	2920.69	2304.18	2401.51	2746.63	2680.74	2717.53 ± 368.5
*Tunica muscularis*	1750.21	1496.11	1378.26	1495.03	1631.72	1630.32	1510.38	1619.36	1618.43	1642.6	1577.24 ± 105.86
*Stratum internum*	1173.18	1000.99	1334.79	1101.11	1556.3	1179.72	1402.83	1489.64	1446.6	1440.42	1312.56 ± 186.36
*Stratum externum*	2923.39	2497.1	2713.05	2596.14	3188.02	2810.04	2913.21	3109	3065.03	3083.02	2889.8 ± 232.08

**Age [Months]**	**Adult**										
**Stomach Wall Layer [µm]**											**mean/sd**
*Epithelium*/*glandulae*	1726.66	1666.22	1542.4	1752.04	1802.92	1749.96	1608.64	1992.14	1882.11	1776.88	1750 ± 129.21
*Tunica mucosa*	2014.68	1895.1	2017.98	1763.89	1810.17	1837.01	2014.45	1887.08	1891.09	1743.99	1887.54 ± 102.08
*Tunica submucosa*	1311.57	975.75	756.89	1379.58	1369.51	1328.52	1223.66	890.86	945.8	1028.19	1121.03 ± 227.18
*Tunica mucosa*/*submucosa*	3326.25	2870.85	2774.87	3143.47	3179.68	3165.53	3238.11	2777.94	2836.89	2772.18	3008.58 ± 220.57
*Tunica muscularis*	1679.78	1915.45	1361.49	1642.53	1573.2	1343.06	1334.12	1979.59	1321.86	1480.6	1563.17 ± 241.32
*Stratum internum*	1764.28	1518.51	1794.35	1727.68	1792.16	1906.24	1932.83	1937.22	1915.37	1939.63	1822.83 ± 133.83
*Stratum externum*	3444.06	3433.96	3155.84	3370.21	3365.36	3249.3	3266.95	3916.81	3237.23	3420.23	3386 ± 210.09

**Table 4:** Histometry of equine stomach—the blind ventricular sac (*saccus caecus*).

**Age [Months]**	**4–5**										
**Stomach Wall Layer [µm]**											**mean/sd**
*Epithelium*/*glandulae*	17.25	21.61	14.07	19.01	16.53	18.55	12.35	19.98	15.7	20.48	17.55 ± 2.94
*Tunica mucosa*	-	-	-	-	-	-	-	-	-	-	-
*Tunica submucosa*	-	-	-	-	-	-	-	-	-	-	-
*Tunica mucosa*/*submucosa*	201.09	222.47	340.48	237.17	294.45	256.72	274.2	254.48	193.33	210.99	248.54 ± 45.75
*Tunica muscularis*	98.64	107.86	66.86	109.97	52.91	128.8	55.8	146.18	97.9	58.52	92.34 ± 32.55
*Stratum internum*	199.78	225.84	196.7	170.43	239.79	283.91	187.53	205.88	156.51	202.76	206.91 ± 36.19
*Stratum externum*	298.42	333.7	263.56	280.4	292.7	412.71	243.33	352.06	254.41	261.28	299.26 ± 52.81

**Age [Months]**	**7–8**										
**Stomach Wall Layer [µm]**											**mean/sd**
*Epithelium*/*glandulae*	116.78	69.99	83.33	101.29	135.22	51.76	55.75	74.59	59.58	102.02	85.03 ± 27.95
*Tunica mucosa*	397.13	349.29	247.78	271.66	364.84	321.77	297.59	225.58	324.12	377.32	317.71 ± 56.8
*Tunica submucosa*	168.12	529.42	326.39	533.49	558.5	449.06	334.59	578.53	592.78	205.49	427.64 ± 158.04
*Tunica mucosa*/*submucosa*	565.25	878.71	574.17	805.15	923.34	770.83	632.18	804.11	916.9	582.81	745.35 ± 144.35
*Tunica muscularis*	419.93	632.39	357.29	494.88	352.68	321.95	361.98	577.11	609.6	558.73	468.65 ± 119.43
*Stratum internum*	1647.28	1731.18	1769.77	1572.05	1622.31	1658.39	1541.54	1792.95	1751.19	1522.66	1660.93 ± 97.36
*Stratum externum*	2067.21	2363.57	2127.06	2066.93	1974.99	1980.34	1903.52	2370.06	2360.79	2081.39	2129.59 ± 174.31

**Age [Months]**	**10–11**										
**Stomach Wall Layer [µm]**											**mean/sd**
*Epithelium*/*glandulae*	418.58	474.76	360.21	413.53	327.73	392.07	327.09	478.67	410.05	347.53	395.02 ± 54.87
*Tunica mucosa*	757.52	1990.46	1226.98	1385.41	1490.39	1491.76	1591.57	1954.74	1934.84	840.34	1466.4 ± 435.61
*Tunica submucosa*	1198.29	1344.96	854.34	1014.76	1104.74	1153.04	1008.88	1132.06	1165.94	1018.04	1099.51 ± 133.55
*Tunica mucosa*/*submucosa*	1955.81	3335.42	2081.32	2400.17	2595.13	2644.8	2600.45	3086.8	3100.78	1858.38	2565.91 ± 504.75
*Tunica muscularis*	1122.89	757.59	956.01	955.33	1315.15	933.49	807.09	1127.16	1129.93	1143.43	1024.81 ± 171.99
*Stratum internum*	864.84	1161.71	1472.97	1202.92	765.28	1177.12	1069.51	1136.64	1472.97	1331.96	1165.59 ± 230.69
*Stratum externum*	1987.73	1919.3	2428.98	2158.25	2080.43	2110.61	1876.6	2263.8	2602.9	2475.39	2190.4 ± 246.36

**Age [Months]**	**Adult**										
**Stomach Wall Layer [µm]**											**mean/sd**
*Epithelium*/*glandulae*	406.5	451.77	337.14	403.74	457.91	372.28	302.4	381.87	574.69	485.03	417.33 ± 78.46
*Tunica mucosa*	904.87	956.45	1028.55	876.43	936.8	931.82	1016.79	947.85	903.25	1061.68	956.45 ± 60.44
*Tunica submucosa*	1013.43	1025.1	1173.33	1169.69	1183.48	994.03	1089.7	1137.79	1167.55	1084.03	1103.8 ± 72.83
*Tunica mucosa*/*submucosa*	1918.3	1981.55	2201.88	2046.12	2120.28	1925.85	2106.49	2085.64	2070.8	2145.71	2060.2 ± 93.37
*Tunica muscularis*	797.14	900.33	533.93	839.81	686.73	931.39	883.56	952.69	679.78	980.59	818.6 ± 143.93
*Stratum internum*	386.41	563.42	554.66	420.06	415.89	319.63	470.54	466.76	557.03	412.97	456.74 ± 81.65
*Stratum externum*	1183.55	1463.75	1088.59	1259.87	1102.62	1251.02	1354.1	1419.45	1236.81	1393.56	1275.3 ± 129.91

**Table 5:** Crown-rump length of equine foetuses.

Age [Months]	4–5	4–5	4–5	4–5	4–5	7–8	7–8	7–8	7–8	7–8	10–11	10–11	10–11	10–11	10–11
CRL [cm]	31.5	34.5	42.1	40.5	52.5	57.1	65.7	64.3	58.9	54	76.1	77.1	75.7	80.1	70

## Data Availability

Not applicable.
